# Primary breast cancer cell culture yields intra-tumor heterogeneous subpopulations expressing exclusive patterns of receptor tyrosine kinases

**DOI:** 10.1186/s12885-016-2769-0

**Published:** 2016-09-20

**Authors:** José Esparza-López, Pier A. Ramos-Elías, Andrea Castro-Sánchez, Leticia Rocha-Zavaleta, Elizabeth Escobar-Arriaga, Alejandro Zentella-Dehesa, Eucario León-Rodríguez, Heriberto Medina-Franco, María de Jesus Ibarra-Sánchez

**Affiliations:** 1 Unidad de Bioquímica, Instituto Nacional de Ciencias Médicas y Nutrición “Salvador Zubirán”, Vasco de Quiroga 15, Belisario Domínguez Sección XVI, Delegación Tlalpan, CP 14080 Distrito Federal Mexico; 2 Departamento de Biología Molecular y Biotecnología, Instituto de Investigaciones Biomédicas, Universidad Nacional Autónoma de México, Circuito Escolar S/N, Ciudad Universitaria, Delegación Coyoacán, CP 04500 Distrito Federal Mexico; 3 Hospital Ángeles del Pedregal, Camino a Santa Teresa # 1055, México, CP 10700 Distrito Federal Mexico; 4 Departamento de Hemato-Oncología, Instituto Nacional de Ciencias Médicas y Nutrición “Salvador Zubirán”, Vasco de Quiroga 15, Belisario Domínguez Sección XVI, Delegación Tlalpan, CP 14080 Distrito Federal Mexico; 5 Departamento de Cirugía, Instituto Nacional de Ciencias Médicas y Nutrición “Salvador Zubirán”, Vasco de Quiroga 15, Belisario Domínguez Sección XVI, Delegación Tlalpan, CP 14080 Distrito Federal Mexico; 6 Departamento de Medicina Genómica y Toxicología Ambiental, Instituto de Investigaciones Biomédicas, Universidad Nacional Autónoma de México, Circuito Escolar S/N, Ciudad Universitaria, Delegación Coyoacán, CP 04500 Distrito Federal Mexico

**Keywords:** Breast cancer, Receptor tyrosine kinases, Intra-tumor heterogeneity, Tyrosine kinase inhibitor, PDGFR

## Abstract

**Background:**

It has become evident that intra-tumor heterogeneity of breast cancer impact on several biological processes such as proliferation, migration, cell death and also might contribute to chemotherapy resistance. The expression of Receptor Tyrosine Kinases (RTKs) has not been analyzed in the context of intra-tumor heterogeneity in a primary breast cancer cell culture. Several subpopulations were isolated from the MBCDF (M serial-breast cancer ductal F line) primary breast cancer cells and were successfully maintained in culture and divided in two groups according to their morphology and RTKs expression pattern, and correlated with biological processes like proliferation, migration, anchorage-independent cell growth, and resistance to cytotoxic chemotherapy drugs and tyrosine kinase inhibitors (TKIs).

**Methods:**

Subpopulations were isolated from MBCDF primary breast cancer cell culture by limiting dilution. RTKs and hormone receptors were examined by Western blot. Proliferation was measure by 3-[4,5-dimethylthiazol-2-yl]-2,5-diphenyl-tetrazolium bromide (MTT assay). Cell viability was evaluated by Crystal Violet. Migration was assessed using Boyden chambers. Anchorage-independent cell growth was evaluated by colony formation in soft agar.

**Results:**

Several subpopulations were isolated from the MBCDF breast cancer cells that were divided into two groups according to their morphology. Analysis of RTKs expression pattern showed that HER1, HER3, c-Met and VEGFR2 were expressed exclusively in cells from group 1, but not in cells from group 2. PDGFR was expressed only in cells from group 2, but not in cells from group 1. HER2, HER4, c-Kit, IGF1-R were expressed in all subpopulations. Biological processes correlated with the RTKs expression pattern. Group 2 subpopulations present the highest rate of cell proliferation, migration and anchorage-independent cell growth. Analysis of susceptibility to chemotherapy drugs and TKIs showed that only Paclitaxel and Imatinib behaved differently between groups. Group 1-cells were resistant to both Paclitaxel and Imatinib.

**Conclusions:**

We demonstrated that subpopulations from MBCDF primary cell culture could be divided into two groups according to their morphology and a RTKs excluding-expression pattern. The differences observed in RTKs expression correlate with the biological characteristics and chemoresistance of each group. These results suggest that intra-tumor heterogeneity contributes to generate groups of subpopulations with a more aggressive phenotype within the tumor.

**Electronic supplementary material:**

The online version of this article (doi:10.1186/s12885-016-2769-0) contains supplementary material, which is available to authorized users.

## Background

Breast cancer is a heterogeneous disease that still is the leading cause of cancer death among women worldwide [1]. Depending on the molecular subtype the clinical outcome is different [2]. Six molecular subtypes are commonly used to determined the course of treatment: luminal A, luminal B, HER2 positive, basal-like, claudin-low and normal-like breast that are determined by the expression of estrogen, progesterone receptor and HER2 [3, 4]. Patients with luminal subtypes benefit from endocrine-directed therapies, while HER2 positive subtype has been associated with poor prognosis. However, HER2-directed therapies have improved the response rates [5].

In recent years, it has become evident that besides the inter-tumor heterogeneity, breast cancer tumors present different subpopulations that can emerge from genetic or epigenetic changes resulting in intra-tumor heterogeneity [6]. Techniques such as cytogenetic analysis, chromosomal hybridization, microarray-based comparative hybridization and massive parallel sequencing have demonstrated that intra-tumor heterogeneity is a common phenomenon in breast cancer [7–11]. Frequent mutations in genes such as *TP53* and *PI3KCA* have been shown by these techniques [12]. Despite all recent advances, intra-tumor heterogeneity is poorly understood, and it still represents the main challenge to judge how representative the analysis of a small biopsy is.

Advances in the understanding of tumor progression have been essential for finding biomarkers that have been useful to determine prognosis as well as targets for drug development. Non-receptor and receptor tyrosine kinases have stood out as putative biomarkers, as is the case of HER2 that has been described as a prognostic and predictive marker for breast cancer. *HER2* gene is amplified in 15–20 % of breast tumors with concomitant HER2 overexpression [13]. Trastuzumab, Pertuzumab and Lapatinib are HER2-directed therapies that have been developed to treat breast cancer [5]. Other RTKs have been associated with poor prognosis in invasive breast carcinomas. The EGFR/HER1 is highly expressed in triple negative compared to other subtypes and it has been associated with endocrine therapy resistance [14, 15]. c-Met is another RTK that is overexpressed in 20–30 % of breast cancer tumors [16, 17]. Association between HER2 and c-Met contributes to resistance to HER2-directed therapy [18]. PDGFRs have also been associated with aggressive breast cancer in advanced stages [19]. PDGFRs expression either in the tumor or the stroma correlates with an aggressive phenotype and poor prognosis [20–22]. RTKs expression has not been analyzed in the context of intra-tumor heterogeneity in breast cancer. In the present work, we isolated subpopulations from a primary breast cancer cell culture; these subpopulations were successfully maintained in culture. We analyzed the RTKs expression pattern and then correlated it with biological processes such as proliferation, migration, and anchorage-independent cell growth as well as the response towards cytotoxic chemotherapy and TKIs. We observed that subpopulations could be divided into two groups according to their morphology and their RTKs pattern. The two groups have an excluding RTKs expression pattern where group 1 expresses HER1, HER3, c-Met and VEGFR2, but it does not express PDGFR, and group 2 express PDGFR, but HER1, HER3, c-Met and VEGFR2 were not present. HER2, HER4, c-Kit, and IGF1-R are present in all subpopulations in variable amounts. PDGFR positive subpopulations have the highest rate of cell proliferation, migration and anchorage-independent cell growth, and they are highly sensitive to Imatinib and Paclitaxel. Other chemotherapy drugs such as Doxorubicin and Capecitabine, as well as Lapatinib and Crizotinib have similar effects on cell viability in all subpopulation tested. These results suggest that the RTKs are expressed in an excluding manner in subpopulations of a heterogeneous breast cancer primary cell culture where the presence of PDGFR confers a more aggressive phenotype. Altogether, these data ratify that breast cancer intra-tumor heterogeneity may contribute to invasion, metastasis and therapy resistance due to different biological characteristics of the subpopulations.

## Methods

### Cell culture

MBCDF primary breast cancer cell culture was previously described [23]. Briefly, a biopsy was obtained from a radical mastectomy from a patient with breast cancer (Protocol approved by the Ethics and Research Committee of the Instituto Nacional de Ciencias Médicas y Nutrición “Salvador Zubirán” (INCMNSZ), Ref. 1549, BQO-008-06/9-1). Written informed consent was obtained from the patient. Tissue was minced and grown as explants in RPMI-1640 plus 10 % fetal bovine serum (FBS). After cells filled the plate, they were trypsinized and grown as a regular cell line. T47D, SK-BR-3 and MCF-7 are from ATCC (Donated by Dr. Rocío Becerra, INCMNSZ). Dr. Alejandro Zentella (INCMNSZ) donated HUVECs.

### Subpopulations isolation by limiting dilution method

MBCDF cells were diluted to 1 cell/200 μl of RPMI-1640 plus 10 % FBS. One hundred microliters were seeded in 96-well plates and grown at 37 ^°^C and 5 % CO_2_. Single colonies were sequentially expanded to 12-well plates, 6-well plates and 100 mm plates. Then subpopulations were grown as regular cell cultures.

### Antibodies

The antibodies against HER2, c-Met and VEGFR were purchased from Cell Signaling Technology (Danvers, MA, USA). The antibodies against estrogen and progesterone receptors were obtained from Cell Marque (Rocklin, CA, USA). The following antibodies were acquired from Santa Cruz Biotechnology (Santa Cruz, CA, USA): HER1, HER3, HER4, IGFI-R, PDGFR.

### Western blotting

Breast cancer cells were lysed in a buffer containing 50 mM HEPES (pH 7.4), 1 mM EDTA, 250 mM NaCl, 1 % Nonidet P-40, 10 mM NaF, 1 mM sodium vanadate and 1× protease inhibitor cocktail (Complete EDTA-free, Roche Diagnostics, Mannheim, Germany). Twenty micrograms of whole protein extract was run in a SDS-PAGE and transferred to Immobilon-P PVDF membrane (Millipore, Bedford, MA, USA). Membranes were blocked with 5 % non-fat milk in PBS-Tween. Membranes were probed with the respective primary antibodies at 4 ^°^C overnight. Secondary HRP-conjugated anti-mouse or anti-rabbit antibodies (Jackson ImmunoResearch, West Grove, PA, USA) were used according to the respective primary antibody. Immunodetection was performed using Supersignal West Pico Chemiluminescent Substrate (Thermo Scientific, Rockford, IL, USA).

### Cell proliferation assay

Breast cancer cells were seeded at a density of 15 000 cells/cm^2^ in 24-well plates in RPMI-1640 supplemented with 10 % FBS. Cell proliferation was quantified by MTT reduction (3-[4,5-dimethylthiazol-2-yl]-2,5-diphenyl-tetrazolium bromide, Sigma-Aldrich, St Louis, MO, USA). Formazan salt was dissolved in acid isopropanol and absorbance was read at 570 and 630 nm in an ELISA plate reader. Results are expressed as the increase in absorbance (570–630 nm) of cells at different days over the absorbance at day 0. Experiments were performed at least three times in triplicate.

### Cell migration assay

Migration assay was performed in a 24-well transwell chamber with 8 μm pore size membranes. MBCDF’s subpopulations were seeded at 30 000 cells in 200 μl of RPMI-1640 plus 10 % FBS in the upper chamber and incubated for 6 h at 37 °C and 5 % of CO_2_. After this time the non-migrating cells in the upper chamber were removed with a cotton swap. Then migrating cells were fixed with 1.1 % of glutaraldehyde in PBS for 20 min and stained with Crystal Violet. Dye excess was removed with water. Number of cells was counted from five fields under the microscope at 20×. Its average was multiplied by viewing field area (0.001 cm^2^) and then multiplied by the Transwell insert area (0.33 cm^2^), giving the total number of migrated cells. To obtain the percentage of migration, the number of migrated cells was divided by the number of seeded cells and then multiplied by 100.

### Soft agar assay

To evaluate anchorage-independent cell growth, an assay of colony formation in soft agar was performed. A bottom layer was formed with 0.5 % agar and RPMI-1640 plus 10 % FBS in 6-well plates. After the bottom layer solidified, the top layer containing 2500 cell/plate, 0.35 % agar and RPMI-1640 plus 10 % FBS was added. Plates were fed every other day for 15 days at 37 °C and 5 % CO_2_. Colonies were stained with 0.005 % Crystal Violet. Experiments were performed at least three independent times in triplicate.

### Cytotoxicity assay

Breast cancer cells were seeded at 10 000 cells/cm^2^ in 48-well plates. Increasing doses of the indicated chemotherapy agents were added and incubated for 48 h at 37 °C and 5 % CO_2_. After this time, cells were fixed for 20 min with 1.1 % glutaraldehyde in PBS, and then stained with Crystal Violet. Dye was dissolved with 10 % acetic acid, and the absorbance was read at 570 nm in an ELISA plate reader. Results are expressed as percentage absorbance at a given concentration over the absorbance of non-treated or vehicle. Experiments were performed three independent times in triplicate.

### Gene silencing by shRNA

MBCDF and its subclone F3 cells were transfected by lipofectamine with four different plasmids pGFP-V-RS containing the following shRNAs sequences specific for PDGFR (Origene Technologies, Rockville, MD, USA):

- 5’ GACGGAGAGTGTGAATGACCATCAGGATG 3’,
- 5’ ACCTTCTCCAGCGTGCTCACACTGACCAA 3’,
- 5’ GAGAGCATCTTCAACAGCCTCTACACCAC 3’,
- 5’ TGCCTCCGACGAGATCTATGAGATCATGC 3’ or irrelevant scramble sequence as negative control. Forty-eight hours after transfection cells were split and plated in presence of 5 μg/ml of Puromycin.

### Statistical analysis

Statistical analysis was performed by GraphPad Prism v_6.0e for MacOs X (GraphPad Software, La Jolla, CA, USA). Significant difference was determined by one-way ANOVA. In TKIs combination assays, we performed Student’s *t* test to evaluate differences between groups. Data were considered statistically significant if *P* < 0.05.

## Results

### Isolation of MBCDF primary breast cancer cells subpopulations

**Fig. 1:**
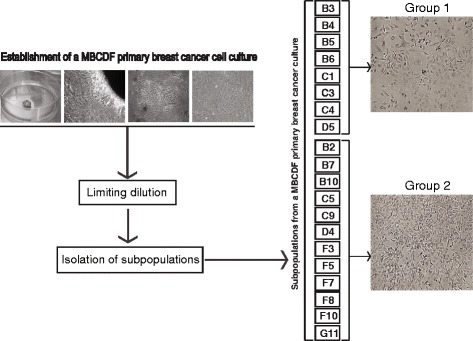
Establishment of a MBCDF primary breast cancer cell culture and subpopulations. Breast tumor biopsy was obtained from a radical mastectomy. Tissue was minced and plated as explants. Cells were grown until they filled the plate. Twenty subpopulations were isolated by limiting dilution and classified into two groups according to their morphology

Previously described MBCDF primary breast cancer cells were cultured by growing explants from a mastectomy biopsy (Fig. 1) [23]. To study breast cancer intra-tumor heterogeneity, subpopulations were isolated from MBCDF primary breast cancer-derived cells by limiting dilution. Twenty different subpopulations were obtained and were classified into two groups according to their morphology. Group 1 has 8 different subpopulations that are characterized by multipolar shape and large cytoplasm (B3, B4, B5, B6, C1, C3, C4 and D5). Group 2 includes 12 subpopulations (B2, B7, B10, C5, C9, D4, F3, F5, F7, F8, F10, G11) with polygonal shape and small cytoplasm (Fig. 1).

### RTKs expression in MBCDF’s subpopulations

**Fig. 2:**
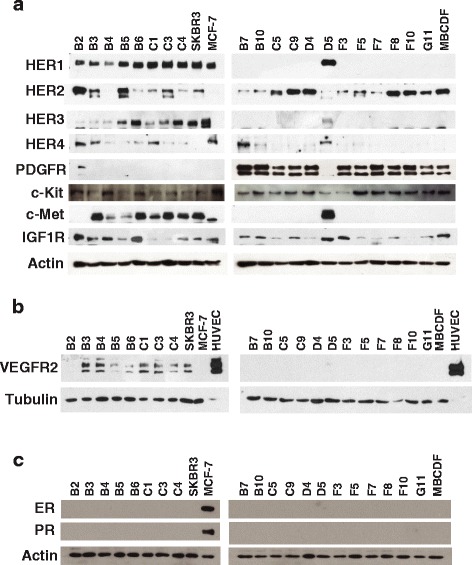
Tyrosine kinase receptors and hormonal receptors expression in MBCDF’s subpopulations. Expression of all receptors was analyzed by Western blot. B2 and D5 were included in their opposite group as controls. Breast cancer cell line SKBR3 was used as control of HER2 expression, and MCF-7 cells were included as a positive control for the expression of ER and PR. **a** HER1, HER2, HER3, HER4, PDGFR, c-Kit, c-Met, IGF1-R expression. Actin was used as loading control. **b** VEGFR2 expression. Protein extracts from HUVECs were used as positive control and Tubulin as loading control. **c** ER and PR receptors expression. Actin was used as loading control

The twenty MBCDF’s subpopulations were characterized by the expression of different RTKs. Using Western blot analysis, we explored the expression of HER1, HER2, HER3, HER4, PDGFR, c-Kit, c-Met, IGF1R, VEGFR2, and hormonal receptors (estrogen and progesterone receptors); SKBR3, MCF-7 cell lines as well as MBCDF were used as control to compare the difference among the parental cells and the subpopulations (Fig. 2). We found an exclusive expression of HER1, HER3, and c-Met in group 1 subpopulations (Fig. 2a, left panel), as well as VEGFR2. The expression of VEGFR2 in the MBCDF’s subpopulations was compared with HUVECs (Fig. 2b, left panel). In the same manner, PDGFR is exclusively expressed in subpopulations from group 2 (Fig. 2a, right panel). HER2, HER4, c-Kit, IGF1-R are indistinctly expressed in all subpopulations. Interestingly, B2 cells were the only subpopulation where HER1, HER3 and PGDFR were expressed together. We did not detect expression of ER or PR in any subpopulation; this is in agreement with the MBCDF negative status for hormonal receptors (Fig. 2c). These results show an intra-tumor heterogeneity in the MBCDF primary breast cancer cell culture, where different subpopulations can be found with a heterogeneous RTKs repertoire with an excluding expression among some of them. Table 1 shows the qualitative amount comparison of the RTKs among the subpopulations.

**Table 1:** Expression of RTKs in MBCDF’s subpopulations

Subclone	HER1	HER2	HER3	HER4	PDGFR	c-Met	IGF-1R	VEGFR2
Group 1								
B5	+++	+++	++	−	−	+	+	−
B6	+++	+	+++	−	−	+++	+++	+
C1	+++	+	++	−	−	++	+	++
C3	+++	++	++	−	−	+++	+	++
C4	+++	+	+++	−	−	++	++	++
D5	+++	+	++	−	−	+	+++	+
B3	++	++	+	+	−	+++	+++	++
B4	+	+	+	−	−	+	+++	++
Group2								
B2	++	+++	+	−	++	−	+++	−
B7	−	+	+	−	+++	−	++	−
B10	−	+	+	−	+++	−	++	−
C5	−	++	+	−	++	−	+	−
C9	−	+++	+	−	+++	−	++	−
D4	−	+++	+	−	++	−	+	−
F3	−	+	+	−	+++	−	+++	−
F5	−	++	+	−	++	−	+	−
F7	−	+	+	−	+	−	+	−
F8	−	+++	+	−	++	−	++	−
F10	−	+++	+	−	++	−	−	−
G11	−	+++	+	−	++	−	+	−

+++ High expression

++ Medium expression

+ Low expression

−Negative expression

### Cell proliferation, cell migration and anchorage-independent cell growth of MBCDF’s subpopulations

**Fig. 3:**
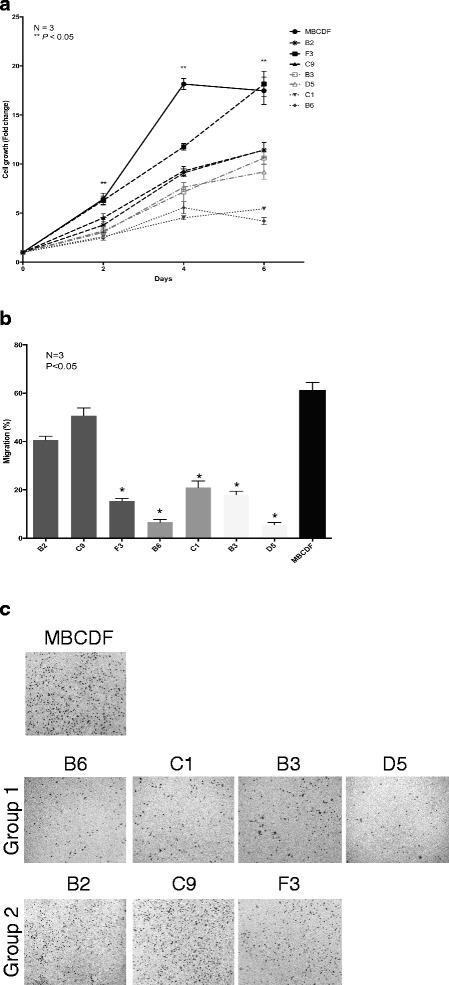
Cell proliferation and migration of MBCDF’s subpopulations. **a** For cell proliferation, B3, B6, C1, D5 (Group 1), B2, F3, C9 (Group 2) subpopulations were seeded at 15 000 cells/cm^2^ in 24-well plate in RPMI plus 10 % FBS. Cell proliferation was evaluated by MTT at the days 0, 2, 4, and 6. Results are presented as the mean ± SEM of three independent experiments seeded in triplicate. ** *P* < 0.05. **b** Migration assays were performed using Boyden chambers. Thirty thousands cells of B3, B6, C1, D5 (Group 1), B2, C9, F3 (Group 2) subpopulations were seeded in the upper chamber in RPMI plus 10 % FBS and incubated for 6 h at 37 °C. After this time the cells that did not migrate were removed from the upper chamber. Cells that migrated were fixed and stained with Cristal Violet. Five fields were counted under the microscope at 20×. **﻿c** Representative pictures of Boyden chamber assays.﻿ The percentage of migrations was calculated as mentioned in Materials and Methods. Migration assays was performed three independent times in triplicate. * *P* < 0.05

To investigate potential biological consequences of the different RTKs expression on the MBCDF’s subpopulation, we evaluated the rates of cell proliferation and cell migration. First, we performed cell proliferation assays on selected subpopulations from each group. We found that F3 cells that belong to group 2 matched the parental MBCDF cells at day 6 at 20 fold. B2 and C9, also from group 2, had an intermediate rate of cell proliferation at 10 fold. B3 and D5 cells from group 1 were slightly below of B2 and C9. Group 1, C1 and B6 subpopulations, had the lowest rate of cell proliferation (Fig. 3a). Our results showed that group 2 subpopulations had a higher capacity to proliferate compared to group 1.

During tumor progression, cells acquire the ability to transmigrate and grow in an anchorage-independent manner. For this reason, we evaluated cell migration by Boyden chamber assay and anchorage-independent cell growth by soft agar assay. In the migration assays, we found that subpopulations from group 2, B2 and C9, migrated the most and there were no significant differences with the MBCDF parental cells. F3 cells, despite being from group 2 showed poor cell migration as well as subpopulations from group 1 (Fig. 3b). Fig. 3c shows representative pictures of Boyden chamber assays.

**Fig. 4:**
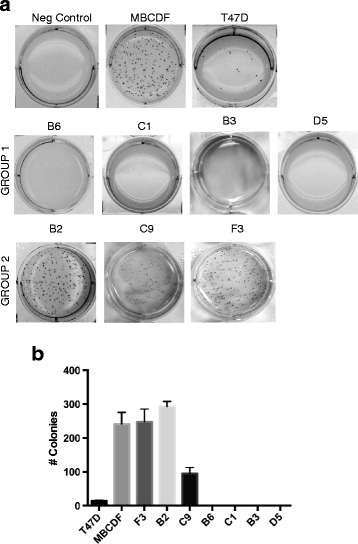
Colony formation of MBCDF’s subpopulations on soft agar. Anchorage-independent cell growth of B3, B6, C1, D5 (Group 1), B2, C9, and F3 (Group 2) subpopulations was evaluated by soft agar assay. A bottom layer of 0.5 % of agar in RPMI plus 10 % FBS was placed. The top layer contained 0.35 % agar in RPMI plus 10 % FBS and 2500 cell/plate. Colonies were analyzed after 15 days of culture. MBCDF and T47D were used as positive controls of colony formation. Agar without cells was used as negative control. **a** Representative picture of colony formation of each subpopulations are presented. **b** Cells were photographed and counted. The graph represents the mean ± SEM. Soft agar assay was performed three independent times in triplicate

In the anchorage-independent colony formation assay, subpopulations from group 2 were capable to grow in soft agar compared with the subpopulations from group 1 that they did not form colonies. B2 and F3 had slightly more number of colonies than MBCDF parental cells. C9 subpopulation formed less and smaller colonies. Interestingly, T47D breast cancer cell line known for its ability to form colonies in soft agar did not show as many colonies as MBCDF cells (Fig. 4a). The number of colonies were counted and graphed: about 292 colonies were generated in B2 cells; F3 and MBCDF had approximately 245 colonies, and C9 had around one hundred colonies. T47D formed an average of 14 colonies (Fig. 4b). Together these data show that the different RTK’s expression pattern influence biological processes such as cells proliferation, migration and anchorage-independent cell growth. In particular, PDGFR expression seems to drive positively these processes.

### Effect of chemotherapy agents on the cell viability of MBCDF’s subpopulations

**Fig. 5:**
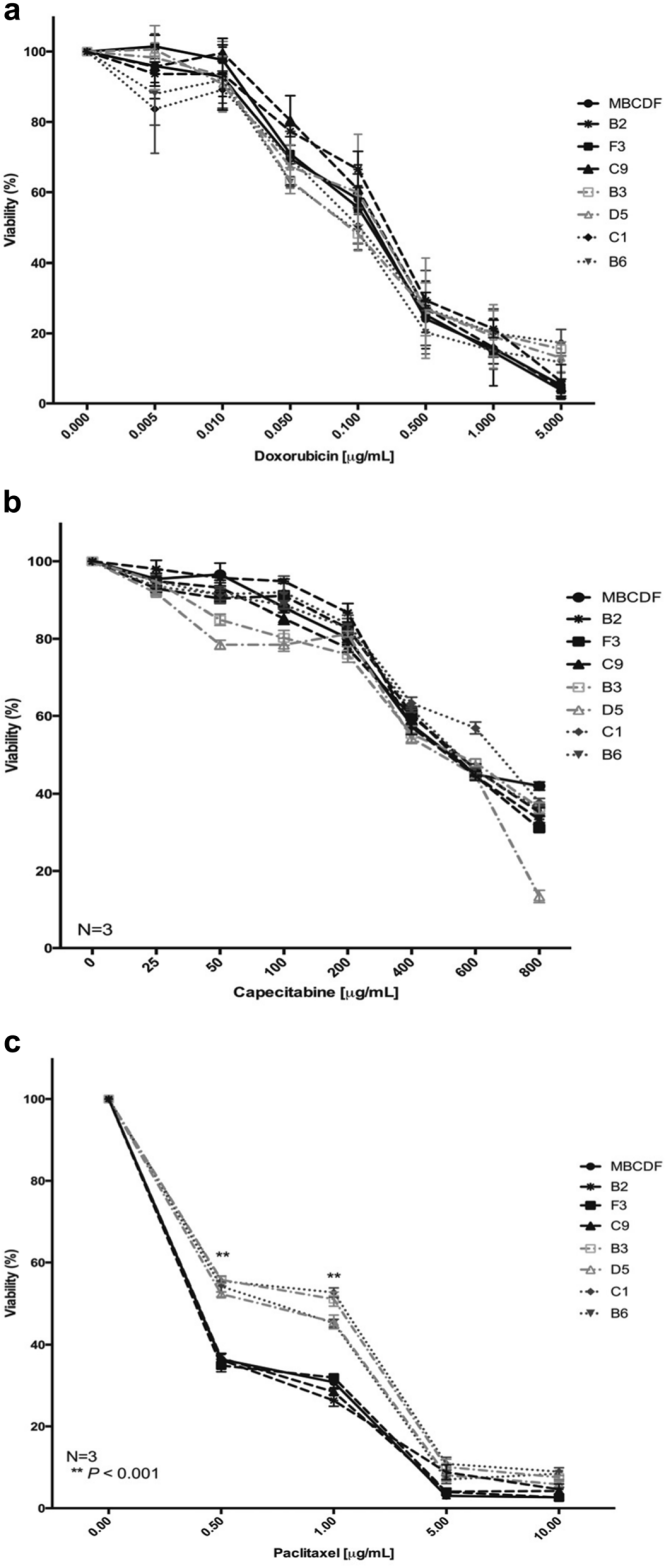
Effect of Doxorubicin, Capecitabine and Paclitaxel on cell viability of MBCDF’s subpopulations. B2, C9, and F3 (Group 2), B3, B6, C1, D5 (Group 1) were seeded at 10 000 cells/cm^2^. **a** Doxorubicin was used at 0, 0.005, 0.01, 0.05, 0.1, 0.5, 1 and 5 μg/mL. **b** Capecitabine was added at 0, 25, 50, 100, 200, 400, 600 and 800 μg/mL. **c** Paclitaxel was added at 0, 0.5, 1, 5 and 10 μg/mL. Viability was evaluated 48 h after addition of the drugs by Crystal Violet assay. Data represent the mean ± SEM of three independent experiments seeded in triplicate. **P < 0.001

Having determined the influence of the RTKs expression pattern on cell proliferation, migration and anchorage-independent cell growth in the MBCDF’s subpopulations, we studied the response of these subpopulations to the chemotherapy agents: Doxorubicin, Capecitabine and Paclitaxel. These drugs are considered cytotoxic: Doxorubicin is an anthracycline that intercalates into the DNA, Capecitabine is an alkylating agent and Paclitaxel is a taxane that stabilize microtubules. We performed cell viability assays with increasing doses of Doxorubicin, Capecitabine and Paclitaxel (Fig. 5). We found that Doxorubicin and Capecitabine treatment induced a decrease on cell viability in a dose-dependent manner; however, these drugs did not show any significant difference among the MBCDF’s subpopulations (Fig. 5a, b). In the case of Paclitaxel treatment, subpopulations from group 1 (B3, D5, C1, B6) were more resistant to its cytotoxic effect. At 0.5 μg/mL of Paclitaxel the cell viability dropped 60 %, remained steady at 1 μg/mL and declined between 5 and 10 % at 5 and 10 μg/mL. Subpopulations from group 2 were more sensitive to Paclitaxel; at 0.5 μg/mL cell viability declined 40 %, continued steady at 1 μg/mL and fell down below 5 % at 5 and 10 μg/mL (Fig. 5c). These data demonstrate that Paclitaxel is the only cytotoxic drug that showed a difference between the two groups of subpopulations.

### Tyrosine kinase inhibitors effect on the cell viability of MBCDF’s subpopulations

**Fig. 6:**
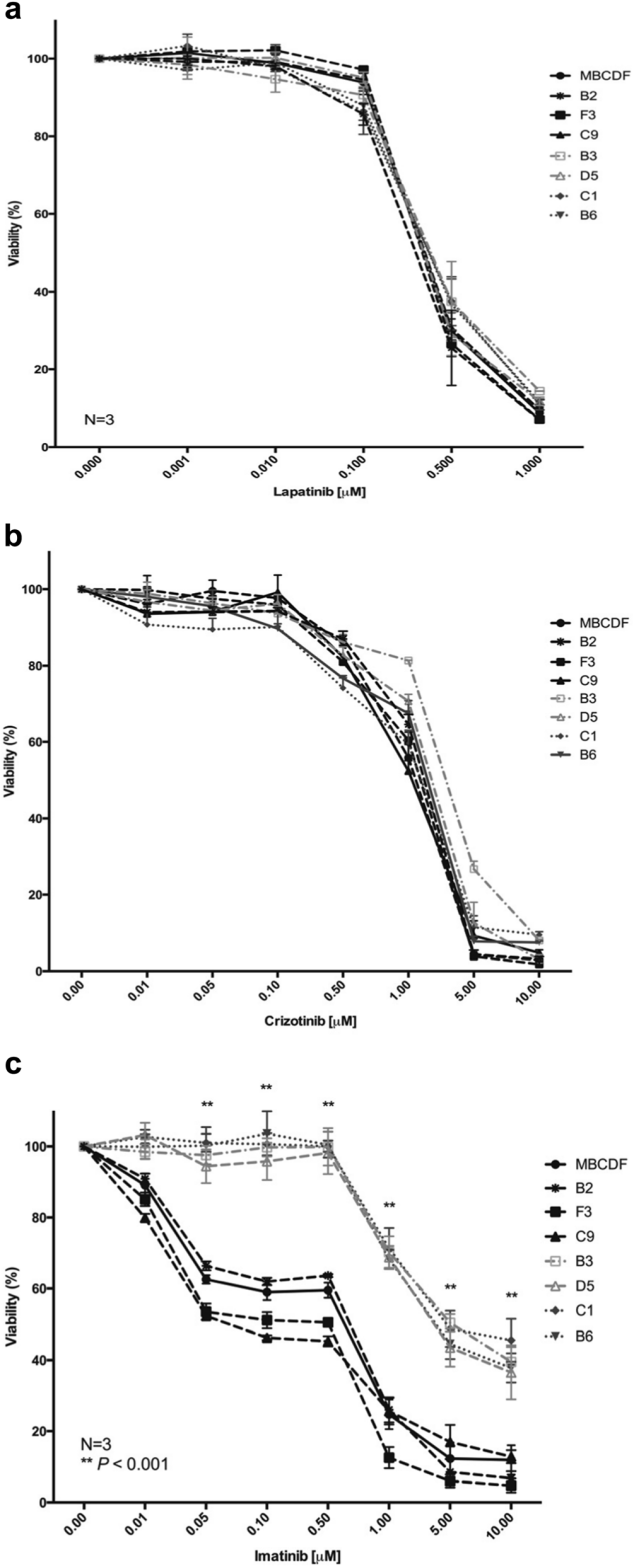
Effect of Lapatinib, Crizotinib and Imatinib on cell viability of MBCDF’s subpopulations. B2, F3, C9 (Group 2), B3, D5, C1, B6 (Group 1) were seeded as in Fig. 5. **a** Lapatinib was used at the following concentrations: 0, 0.001, 0.01, 0.1, 0.5 and 1 μM. **b** Crizotinib was added at 0, 0.01, 0.05, 0.1, 0.5, 1, 5 and 10 μM. **c** The following concentrations were used for Imatinib: 0, 0.01, 0.05, 0.1, 0.5, 1, 5 and 10 μM. Cell viability was evaluated as in Fig. 5. Data represent the mean ± SEM of three independent experiments seeded in triplicate. **P < 0.001

Since we observed a RTKs excluding pattern between group 1 and group 2, we analyzed whether the RTKs distribution influence the susceptibility to tyrosine kinase inhibitors. We did viability assays as in Fig. 5, but in this case using Lapatinib, a HER1 and HER2 inhibitor; Crizotinib that targets c-Met and Alk; and Imatinib, an inhibitor of PDGFR, Abl and c-Kit. Treatment with either Lapatinib or Crizotinib did not show significant differences between the two groups of subpopulations (Fig. 6a, b). MBCDF subpopulations showed significant difference to Imatinib treatment. Subpopulations from group 1 presented marked resistance to Imatinib from 0.01 to 0.5 μM, then cell viability declined in dose dependent manner from 1 to 10 μM. In the case of subpopulations from group 2 treated with Imatinib, cell viability declined in dose dependent manner from 0.01 to 0.05 μM, remaining steady up to 0.5 μM, and cell viability dropped below 15 % (Fig. 6c). These data demonstrate that some RTKs influence the response to TKIs; in particular, PDGFR expression sensitizes breast cancer cells to Imatinib. In order to confirm this hypothesis, we silenced the expression of PDGFR gene in MBCDF and F3 with specific shRNA. PDGFR expression was significantly reduced after transfection with specific PDGFR shRNAs, but not after transfection with a control scramble shRNAs. As expected treatment with increasing doses of Imatinib induced a significant decrease on viability of both MBCDF sh control and F3 sh control cells transfected with the irrelevant shRNA. However, silencing of PDGFR resulted in partial resistance to Imatinib (Additional file 1: Fig. S1).

### Effect of combinations of tyrosine kinase inhibitors on MBCDF’s subpopulations viability

**Fig. 7:**
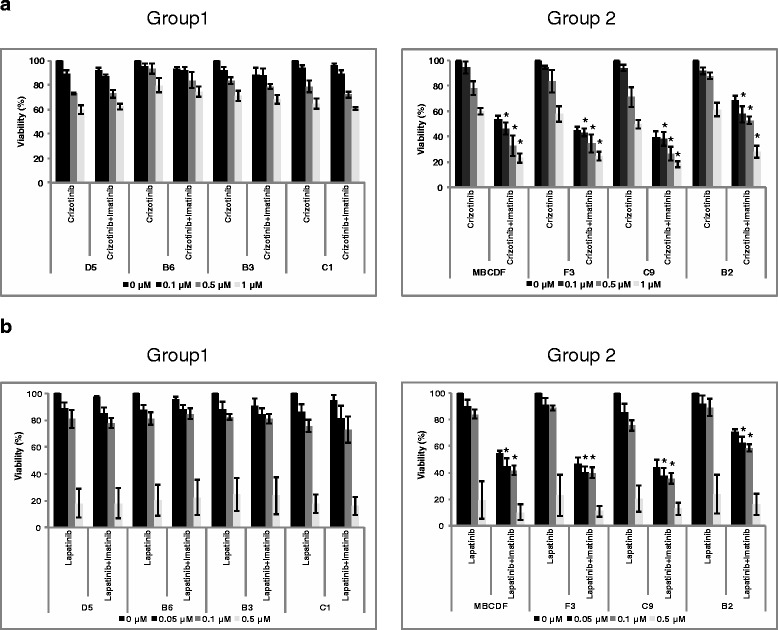
Effect of the combinations Crizotinib-Imatinib, and Lapatinib-Imatinib on cell viability. MBCDF and its subpopulations: D5, B6, B3, C1 (Group 1, left panel), F3, C9, B2 (Group 2, right panel) were seeded as in Fig. 5. **a** Crizotinib was used at the following concentrations: 0, 0.1, 0.5 and 1 μM with Imatinib 1 μM. **b** Lapatinib was added at 0, 0.05, 0.1, 0.5 μM plus Imatinib 1 μM. Cell viability was evaluated as in Fig. 5. Data represent the mean ± SEM of three independent experiments seeded in triplicate. * *P* < 0.05 *versus* Crizotinib or Lapatinib alone

In order to explore a putative translational implication of inhibition of RTKs in MBCDF’s subpopulations, we treated the cells with a combination of Crizotinib with Imatinib and Lapatinib with Imatinib. First, we treated subpopulations from group 1 and group 2 with increasing doses of Crizotinib (0, 0.1, 0.5 and 1 μM) and a fixed dose of Imatinib (1 μM). We found that treatment with Crizotinib-Imatinib had no further cytotoxic effect than Crizotinib alone in cells from group 1. However, Crizotinib-Imatinib combination induced an increment in cell death on group 2 cells with a general additive effect (Fig. 7a). Next, we evaluated a combination of Lapatinib-Imatinib in MBCDF’s subpopulations. We used increasing doses of Lapatinib (0, 0.05, 0.1 and 0.5 μM) and a fixed dose of Imatinib (1 μM). We found similar results to those obtained with the combination of Crizotinib-Imatinib, where in group 1 cells the effect of Lapatinib was not improved by the addition of Imatinib. Nevertheless, Lapatinib-Imatinib combination increased the cell death of group 2 cells (Fig. 7b). These data suggest that a putative translational used of these TKIs depend on the expression pattern of RTKs.

## Discussion

In this work, we isolated and maintained several subpopulations from a primary breast cancer cell culture (MBCDF) [23]. The subpopulations were divided into two groups according to their morphology. The analysis of RTKs expression was correlated with biological processes such as cell proliferation, migration and anchorage-independent cell growth. We also linked each group with the susceptibility to cytotoxic chemotherapy drugs and TKIs. The data presented in this study ratifies breast cancer tumor heterogeneity as has been shown before [24–26], and this is the first time that subpopulations from a primary breast cancer cells are successfully maintained in cell culture with a stable phenotype.

The concept of tumor heterogeneity is well established in cancer research [24–27], and several studies have addressed breast cancer tumor heterogeneity. These works most of the times have been searching for gene amplification [26, 28–30]. Few studies have analyzed the relation among some RTKs such as members of the HER family with c-Met [18]. We analyzed a broader number of RTKs that showed an excluding expression pattern. Overall, we found that group 1 expresses solely HER1, HER3, c-Met and VEGFR2, but not PDGFR that is limited to group 2. Other RTKs (HER2, HER4, c-Kit and IGF-1R) have variable expression with no significant pattern among subpopulations. It is well established that RTKs regulate biological processes and are submitted to major regulatory mechanisms. Here, we found a correlation between the RTKs expression and biological processes such as proliferation, migration and anchorage-independent cell growth. Subpopulations from group 1 have poor cell proliferation, migration and anchorage-independent cell growth. Interestingly, expression of PDGFR in subpopulations from group 2 correlates with high cell proliferation, migration and anchorage-independent cell growth; these results suggest that PDGFR might play an important role in these biological processes. Our results agree with a recent report showing that inhibition of PDGFR with TKIs inhibits cell proliferation and migration of breast cancer cell lines [31]. Furthermore, the expression of PDGFR in the stroma and cancer cells has been associated with poor prognosis of breast cancer patients [20–22].

Despite the advances in breast cancer treatment, the mortality of this disease is still high due to the development of resistance to either cytotoxic therapy or targeted therapy. Heterogeneous tumors may contain subclones with either intrinsic or acquired resistance. Furthermore, subpopulations under selective pressure by treatment can give rise to new cancer cells with the potential to drive progression of the disease [32]. We analyzed the effect of several cytotoxic and targeted therapy agents on the MBCDF subclones. Three cytotoxic drugs (Doxorubicin, Capecitabine and Paclitaxel) and three targeted therapies (Lapatinib, Crizotinib and Imatinib) were tested. Paclitaxel and Imatinib were the only drugs that presented significant differences between the two groups. Group 1 was resistant to Paclitaxel and Imatinib; an opposite effect was observed in subclones from group 2, sensitive to Paclitaxel and Imatinib. Cell reprogramming through an epithelial-mesenchymal transition (EMT) program might be a putative mechanism of Paclitaxel resistance, since there is evidence that this process contributes to metastasis as well as drug resistance [33, 34]. Further studies remain to be performed to determine the Paclitaxel resistance mechanism in cells from group 1.

Imatinib resistance in group 1 subclones could be explained by the lack of PDGFR. We proved that silencing of PDGFR by shRNAs induced partial resistance to Imatinib. We have previously demonstrated that the sensitivity to Imatinib relies also in part on c-Abl kinase in breast cancer cell lines as well as primary cell culture MBCDF [23]. A putative translational implication of the diverse RTKs expression pattern described here would be co-targeting the different types of subpopulations in a tumor. We explored the combination of TKIs that target RTKs from each group. Interestingly, the combination of Crizotinib with Imatinib or Lapatinib-Imatinib had an additive effect only on group 2 subpopulations, but no in the cells from group 1, where it was only observed the effect of Crizotinib or Lapatinib alone. The presence of PDGFR in group 2 cells seems to be important to increase cell death with these combinations. Also, these combinations might be potentially useful in the treatment of breast cancer patients that have combined expression of RTKs such as PDGFR, c-Met and HER family members. It is clear that group 1 cells are resistant to TKIs either alone or in combination, for this reason it is necessary to find a better combination either of TKIs or TKIs with classical chemotherapy to improve cell death on group 1 cells.

MBCDF primary cell culture is classified as HER2+; most of its subpopulations present different amounts of HER2, except the subclone B4 that can be catalogued as a triple negative. Another interesting subclone is B2 that co-expressed PDGFR with HER1, HER3, c-Met and VEGFR2; despite expressing group 1 receptors, B2 cells behave mainly as group 2 subpopulation with high proliferation, migration, anchorage-independent cell growth, as well as sensitivity to Paclitaxel and Imatinib. The data from B2 cells support a major role of PDGFR in the biological processes studied. Moreover, HER2, HER4, c-Kit and IGF-1R are expressed in different amounts without any specific correlation; however, these RTKs might participate in the resistance to chemotherapy. For example, expression of IGF1-R has been associated with resistance to Trastuzumab [35, 36]. Further studies need to be done to determine putative crosstalk among these RTKs.

## Conclusions

These report demonstrate intra-tumor heterogeneity in the MBCDF primary breast cancer cell culture. MBCDF is composed of several subpopulations with different RTKs profile. Some of the RTKs showed an excluding pattern among the subpopulations. Breast cancer subpopulations with a particular expression of RTKs correlate with biological processes such as proliferation, migration and anchorage-independent cell growth and response to chemotherapy agents. The intra-tumor heterogeneity observed in the MBCDF primary breast cancer cell culture suggests that subpopulations with a specific RTKs repertoire may have a more aggressive phenotype within the tumor. These results open the door to address new schemes of treatment for breast cancer patients focusing in the RTK pattern of tumor subpopulations.

## Additional file

Additional file 1: Fig. S1. Effect of PDGFR silencing on Imatinib-induced cytotoxicity. MBCDF (a) and subclone F3 (b) cells were transfected with either specific PDGFR shRNAs (sh PDGFR) or scrambled shRNAs (sh control). Transfected cells were selected with 5 μg/ml of Puromycin. Selected cells were treated with the indicated doses of Imatinib for 48 h. Cell viability was evaluated by Crystal Violet assay (left panel). Silencing of PDGFR expression was corroborated by Western blot (right panel). Data represent the mean ± SEM of three independent experiments seeded in triplicate. * *P* < 0.05 *versus* sh control (EPS 4216 kb)

## Abbreviations

c-Kit: Stem cell factor receptor
c-Met: Hepatocyte growth factor receptor
FBS: Fetal bovine serum
HER1/EGFR: Epidermal growth factor receptor
HER2: Epidermal growth factor receptor 2
HER3: Epidermal growth factor receptor 3
HER4: Epidermal growth factor receptor 4
HUVECs: Human umbilical vein cells
IGF1-R: Insulin-like growth factor-1 receptor
PBS: Phosphate-buffered saline
PDGFR: Platelet-derived growth factor receptor
PI3KCA: Class A phosphoinositide 3-kinase
RTK: Receptor tyrosine kinase
TP53: Tumor protein p53
VEGFR2: Vascular endothelial growth factor receptor 2

## References

[1] Ferlay J, Soerjomataram I, Dikshit R, Eser S, Mathers C, Rebelo M (2015). Cancer incidence and mortality worldwide: sources, methods and major patterns in GLOBOCAN 2012. Int J Cancer J Int cancer.

[2] Perou CM, Jeffrey SS, van de Rijn M, Rees CA, Eisen MB, Ross DT (1999). Distinctive gene expression patterns in human mammary epithelial cells and breast cancers. Proc Natl Acad Sci U S A.

[3] Perou CM, Sorlie T, Eisen MB, van de Rijn M, Jeffrey SS, Rees CA (2000). Molecular portraits of human breast tumours. Nature.

[4] Prat A, Perou CM (2011). Deconstructing the molecular portraits of breast cancer. Mol Oncol.

[5] Slamon D, Eiermann W, Robert N, Pienkowski T, Martin M, Press M (2011). Adjuvant trastuzumab in HER2-positive breast cancer. N Engl J Med.

[6] Marusyk A, Almendro V, Polyak K (2012). Intra-tumour heterogeneity: a looking glass for cancer?. Nat Rev Cancer.

[7] Ding L, Ellis MJ, Li S, Larson DE, Chen K, Wallis JW (2010). Genome remodelling in a basal-like breast cancer metastasis and xenograft. Nature.

[8] Navin N, Krasnitz A, Rodgers L, Cook K, Meth J, Kendall J (2010). Inferring tumor progression from genomic heterogeneity. Genome Res.

[9] Martelotto LG, Ng CK, Piscuoglio S, Weigelt B, Reis-Filho JS (2014). Breast cancer intra-tumor heterogeneity. Breast Cancer Res.

[10] Cottu PH, Asselah J, Lae M, Pierga JY, Dieras V, Mignot L (2008). Intratumoral heterogeneity of HER2/neu expression and its consequences for the management of advanced breast cancer. Ann Oncol.

[11] Turner NC, Reis-Filho JS (2012). Genetic heterogeneity and cancer drug resistance. Lancet Oncol.

[12] Shah SP, Roth A, Goya R, Oloumi A, Ha G, Zhao Y (2012). The clonal and mutational evolution spectrum of primary triple-negative breast cancers. Nature.

[13] Slamon DJ, Clark GM, Wong SG, Levin WJ, Ullrich A, McGuire WL (1987). Human breast cancer: correlation of relapse and survival with amplification of the HER-2/neu oncogene. Science.

[14] Massarweh S, Osborne CK, Creighton CJ, Qin L, Tsimelzon A, Huang S (2008). Tamoxifen resistance in breast tumors is driven by growth factor receptor signaling with repression of classic estrogen receptor genomic function. Cancer Res.

[15] Hoadley KA, Weigman VJ, Fan C, Sawyer LR, He X, Troester MA (2007). EGFR associated expression profiles vary with breast tumor subtype. BMC Genomics.

[16] Ghoussoub RA, Dillon DA, D’Aquila T, Rimm EB, Fearon ER, Rimm DL (1998). Expression of c-met is a strong independent prognostic factor in breast carcinoma. Cancer.

[17] Edakuni G, Sasatomi E, Satoh T, Tokunaga O, Miyazaki K (2001). Expression of the hepatocyte growth factor/c-Met pathway is increased at the cancer front in breast carcinoma. Pathol Int.

[18] Paulson AK, Linklater ES, Berghuis BD, App CA, Oostendorp LD, Paulson JE (2013). MET and ERBB2 are coexpressed in ERBB2+ breast cancer and contribute to innate resistance. Mol Cancer Res MCR.

[19] Seymour L, Dajee D, Bezwoda WR (1993). Tissue platelet derived-growth factor (PDGF) predicts for shortened survival and treatment failure in advanced breast cancer. Breast Cancer Res Treat.

[20] Bhardwaj B, Klassen J, Cossette N, Sterns E, Tuck A, Deeley R (1996). Localization of platelet-derived growth factor beta receptor expression in the periepithelial stroma of human breast carcinoma. Clin Cancer Res.

[21] Coltrera MD, Wang J, Porter PL, Gown AM (1995). Expression of platelet-derived growth factor B-chain and the platelet-derived growth factor receptor beta subunit in human breast tissue and breast carcinoma. Cancer Res.

[22] Carvalho I, Milanezi F, Martins A, Reis RM, Schmitt F (2005). Overexpression of platelet-derived growth factor receptor alpha in breast cancer is associated with tumour progression. Breast Cancer Res.

[23] Esparza-Lopez J, Medina-Franco H, Escobar-Arriaga E, Leon-Rodriguez E, Zentella-Dehesa A, Ibarra-Sanchez MJ. Doxorubicin induces atypical NF-kappaB activation through c-Abl kinase activity in breast cancer cells. Journal of cancer research and clinical oncology. 2013. doi:10.1007/s00432-013-1476-3. 10.1007/s00432-013-1476-3 PMC11824581 23892407

[24] Metzger-Filho O, Tutt A, de Azambuja E, Saini KS, Viale G, Loi S (2012). Dissecting the heterogeneity of triple-negative breast cancer. J Clin Oncol Off J Am Soc Clin Oncol.

[25] Norton KA, Popel AS, Pandey NB (2015). Heterogeneity of chemokine cell-surface receptor expression in triple-negative breast cancer. Am J Cancer Res.

[26] Polyak K (2011). Heterogeneity in breast cancer. J Clin Invest.

[27] Janiszewska M, Beca F, Polyak K (2014). Tumor heterogeneity: the Lernaean hydra of oncology?. Oncology.

[28] Shipitsin M, Campbell LL, Argani P, Weremowicz S, Bloushtain-Qimron N, Yao J (2007). Molecular definition of breast tumor heterogeneity. Cancer Cell.

[29] Denisov EV, Litviakov NV, Zavyalova MV, Perelmuter VM, Vtorushin SV, Tsyganov MM (2014). Intratumoral morphological heterogeneity of breast cancer: neoadjuvant chemotherapy efficiency and multidrug resistance gene expression. Sci Rep.

[30] Pekar G, Gere M, Tarjan M, Hellberg D, Tot T (2014). Molecular phenotype of the foci in multifocal invasive breast carcinomas: intertumoral heterogeneity is related to shorter survival and may influence the choice of therapy. Cancer.

[31] Stalker L, Pemberton J, Moorehead RA (2014). Inhibition of proliferation and migration of luminal and claudin-low breast cancer cells by PDGFR inhibitors. Cancer Cell Int.

[32] Mcgranahan N, Swanton C (2015). Biological and therapeutic impact of intratumor heterogeneity in cancer evolution. Cancer Cell.

[33] Gurzu S, Turdean S, Kovecsi A, Contac AO, Jung I (2015). Epithelial-mesenchymal, mesenchymal-epithelial, and endothelial-mesenchymal transitions in malignant tumors: An update. World j clin cases.

[34] Huang J, Li H, Ren G (2015). Epithelial-mesenchymal transition and drug resistance in breast cancer (Review). Int J Oncol.

[35] Browne BC, Eustace AJ, Kennedy S, O’Brien NA, Pedersen K, McDermott MS (2012). Evaluation of IGF1R and phosphorylated IGF1R as targets in HER2-positive breast cancer cell lines and tumours. Breast Cancer Res Treat.

[36] Jin Q, Esteva FJ (2008). Cross-talk between the ErbB/HER family and the type I insulin-like growth factor receptor signaling pathway in breast cancer. J Mammary Gland Biol Neoplasia.

